# Microsatellites versus single-nucleotide polymorphisms in linkage analysis for quantitative and qualitative measures

**DOI:** 10.1186/1471-2156-6-S1-S122

**Published:** 2005-12-30

**Authors:** Gerald Dunn, Anthony L Hinrichs, Sarah Bertelsen, Carol H Jin, John SK Kauwe, Brian K Suarez, Laura J Bierut

**Affiliations:** 1 Department of Psychiatry, Genetics, Washington University School of Medicine, St. Louis, Missouri; 2 Department of Genetics, Washington University School of Medicine, St. Louis, Missouri

## Abstract

**Background:**

Genetic maps based on single-nucleotide polymorphisms (SNP) are increasingly being used as an alternative to microsatellite maps. This study compares linkage results for both types of maps for a neurophysiology phenotype and for an alcohol dependence phenotype. Our analysis used two SNP maps on the Illumina and Affymetrix platforms. We also considered the effect of high linkage disequilibrium (LD) in regions near the linkage peaks by analysing a "sparse" SNP map obtained by dropping some markers in high LD with other markers in those regions.

**Results:**

The neurophysiology phenotype at the main linkage peak near 130 MB gave LOD scores of 2.76, 2.53, 3.22, and 2.68 for the microsatellite, Affymetrix, Illumina, and Illumina-sparse maps, respectively. The alcohol dependence phenotype at the main linkage peak near 101 MB gave LOD scores of 3.09, 3.69, 4.08, and 4.11 for the microsatellite, Affymetrix, Illumina, and Illumina-sparse maps, respectively.

**Conclusion:**

The linkage results were stronger overall for SNPs than for microsatellites for both phenotypes. However, LOD scores may be artificially elevated in regions of high LD. Our analysis indicates that appropriately thinning a SNP map in regions of high LD should give more accurate LOD scores. These results suggest that SNPs can be an efficient substitute for microsatellites for linkage analysis of both quantitative and qualitative phenotypes.

## Background

Microsatellites are commonly used as markers for linkage analysis. More recently, single-nucleotide polymorphisms (SNPs) have been increasingly used as genotyping markers due in part to lower cost and ease of use. This study compares SNP marker linkage results for qualitative and quantitative phenotypes to the corresponding microsatellite results. For this study we focused on chromosome 7, an area shown to have linkage and association for both alcohol dependence, a qualitative trait, and a neurophysiologic measure, a quantitative trait [1-4]. For the neurophysiologic measure, evidence of linkage and association has been demonstrated for chromosome 7 [2]. Alcohol dependence was examined by Foroud et al. [1] for evidence of linkage relative to two populations. The results of that study indicate evidence of linkage only on chromosome 7 for the samples both individually and combined. Because the Genetic Analysis Workshop 14 (GAW14) population is a mixture of these two samples, we restricted our analyses to chromosome 7.

## Methods

### Sample

The GAW14 population data consisted of phenotypic and genotypic information for 1,614 individuals in 143 pedigrees. Because of a significant difference in the allele frequencies for African Americans and Caucasians in this population, our analyses were restricted to the larger Caucasian subpopulation of 1,214 individuals in 112 pedigrees [5].

### Analyses

For the alcohol dependence phenotype an individual was defined as affected by DSM-III-R alcohol dependence and Feighner definite alcoholism. For this phenotype, an affected sibling pair method was used using GENEHUNTER, where only individuals who are affected are analyzed, though unaffected individuals can contribute information regarding IBD sharing. We used the *n*-1 sib pair method with the proband being matched to all other affected siblings. This strategy was chosen because the proband is generally more severely affected and more likely represents a "true" case.

We used a new version of GENEHUNTER [6], which has recently been released to deal with SNP markers. Even with these recent enhancements, the complete Affymetrix map with 578 markers on chromosome 7 still surpassed the memory capacity of our systems. We therefore constructed three separate maps consisting of the first 289 markers, the last 289 markers and 289 markers from the middle of chromosome 7. The composite map consisted of the first two-thirds of the first set, the middle two-thirds of the middle set and the last two-thirds of the last set. This ensures that all markers in the composite map have at least some multipoint support from flanking markers. Once the chromosome was divided, the data files could be loaded. Some of the large pedigrees created prohibitive computational time requirements. We set the MAXBITS = 16 option in GENEHUNTER to allow it to automatically remove individuals from the pedigrees to reduce the large pedigrees to manageable form. In almost every case only unaffecteds were trimmed.

The neurophysiology phenotype was the target case frontal theta band [2], denoted ttth1 in the GAW14 data. The linkage analysis was carried out using SOLAR [7]. We screened for age and sex covariates; only age was significant (*p *= 2 × 10^-14^).

Multipoint IBD matrices were computed at 1-cM intervals using LOKI and output into SOLAR format. Two-point identity-by-descent (IBD) values were computed using SOLAR.

It was found by Hinrichs et al. [8] that a subset of the Illumina map obtained by removing markers in high linkage disequilibrium (LD) with nearby markers provided nearly as much information as the full map and more information than the microsatellite map. In view of this, we also ran the linkage analysis using this sparser map. The marker densities for the four maps were: microsatellites, 1 per 6.03 cM; Affymetrix, 1 SNP per 0.31 cM; Illumina, 1 SNP per 0.68 cM; and sparse Illumina, 1 SNP per 1.10 cM.

Although genetic maps were provided and were used for IBD computations and linkage analyses, chromosome lengths for microsatellites, Affymetrix SNPs, and Illumina SNPs were different (187 cM, 178 cM, and 185 cM) and therefore markers were adjusted to their physical locations for the purpose of plotting and ease of comparison. All findings were then placed on a common map defined by the physical map obtained from the NCBI database (Build 34.3).

## Results

**Figure 1:**
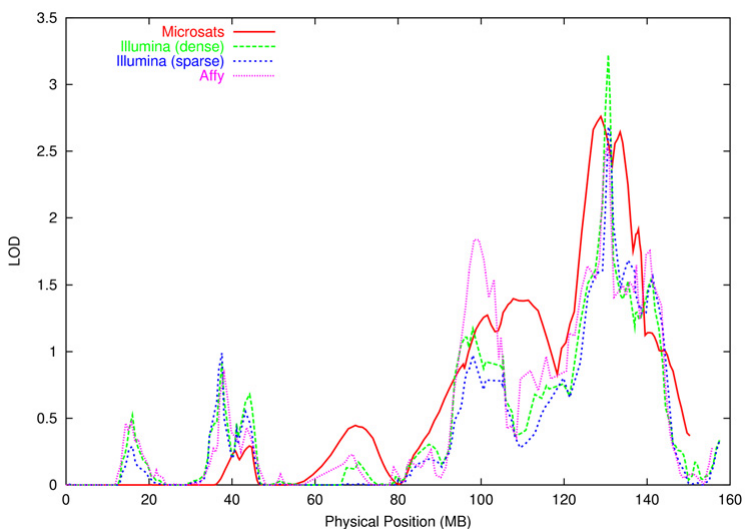
Multipoint linkage analyses for the neurophysiology phenotype.

**Figure 2:**
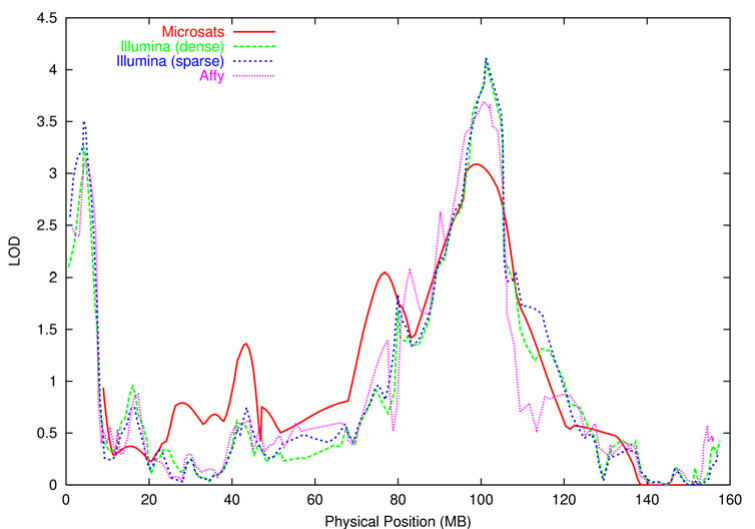
Multipoint linkage analyses for the alcohol dependence phenotype.

In the present analyses, linkage results were stronger overall for SNPs than for microsatellites. We found higher LOD scores and more narrowly defined linkage peaks with SNPs for both the quantitative and qualitative phenotypes (Figures 1 and 2). For the neurophysiology phenotype at the main linkage peak near 130 MB, the Illumina and Affymetrix markers produced LOD scores of 3.22 and 2.53, respectively, and the microsatellite markers gave a LOD score of 2.76. Using the sparse Illumina map the LOD score was 2.68. For the alcohol dependence phenotype at the linkage peak near 101 MB, the Illumina and Affymetrix markers gave LOD scores of 4.08 and 3.69, while the microsatellite markers had a LOD score of 3.09. The sparse Illumina map gave a LOD score of 4.11.

In order to interpret our linkage results, we compared the dense and sparse Illumina maps near the two linkage peaks. There were 19 markers within 5 MB of the peak near 130 MB and 7 of these were dropped for the sparse map. There were 17 markers within 5 MB of the peak near 101 MB and 2 of these were dropped for the sparse map.

We also performed a two-point analysis for the neurophysiology phenotype at the linkage peak near 130 MB. The Illumina SNPs produced a LOD score of 1.65 and the microsatellite markers gave a LOD score of 2.32.

## Discussion

These analyses provide a comparison of the use of microsatellite and SNP markers in linkage analysis for quantitative and qualitative phenotypes. For both phenotypes linkage results were stronger overall for SNPs than for microsatellites. Does this represent a true or a false increase in the evidence of linkage? The SNP maps have higher information content than microsatellite maps [8], and this may contribute to the higher LOD scores. However, there is also substantial pair-wise LD throughout the chromosome. Ignoring this and treating each SNP as an isolated marker may erroneously elevate sharing estimates. For the alcohol dependence phenotype, the LOD scores obtained using the dense and sparse Illumina maps were virtually the same, indicating that these scores were not artificially elevated by the LD near this location (only 2 of 17 markers were dropped for the sparse map). For the neurophysiology phenotype, the LOD scores using the microsatellite, Affymetrix, and sparse Illumina maps were nearly the same. The dense Illumina map gave a marginally higher LOD score, and this may reasonably be attributed to high LD in this region (7 of 19 markers were dropped for the sparse map). Although the LOD scores varied more with the quantitative trait than with the qualitative trait for the two Illumina maps, this may be due more to LD in the region than to the different trait types. These results indicate that a sparse map as constructed in [8] can carry sufficient information for linkage.

As one expects, the two-point analysis of the neurophysiology phenotype gave lower linkage signals than the multipoint analysis with a much greater difference evident with SNPs than with microsatellites. Such differences are often exaggerated in regions of high LD. In addition, it is known that single-point analysis with SNPs has substantially lower information than with microsatellites. In this case we believe that the differing linkage results are more due to reduced information in the SNPs than to LD in the region.

There was also some difference in LOD scores between the Affymetrix and Illumina SNPs, with the Illumina SNPs giving higher scores at the main linkage peaks in these analyses. These differences may be due to the differing number of SNPs typed and their distribution on the chromosome. Though the overall information content for the Illumina and Affymetrix maps were similar, by chance it appears that the information content is lower in the Affymetrix map at these linkage peaks.

## Conclusion

Our results indicate that SNPs can perform as well as or better than microsatellites for linkage analysis. In conjunction with the results of [8], it seems wise to thin a SNP map in regions of high LD to avoid artificially high LOD scores. Overall, our analyses suggest that SNPs are an efficient substitute for microsatellites for linkage analysis of both quantitative and qualitative phenotypes.

## Abbreviations

GAW14: Genetic Analysis Workshop 14

IBD: Identity-by-descent

LD: Linkage disequilibrium

SNP: Single-nucleotide polymorphism

## Authors' contributions

GD, SB, and ALH formatted the data files and analyzed the data. GD, LJB and ALH drafted the manuscript. JSKK provided analyses identifying Caucasian subgroups. GD, SB, ALH, LJB, CHJ, and BKS participated in the design and coordination of the study. All authors read and approved the final manuscript.
